# Development and validation of evaluation indicators for the quality of professional life of Chinese homeroom teachers

**DOI:** 10.3389/fpsyg.2025.1650566

**Published:** 2025-12-05

**Authors:** Li Sun, Ao Li, ShuQin Li, QingXiu Liu, JingPing Lu

**Affiliations:** 1 School of Education and Science, Hubei University of Education, Wuhan, China; 2 SSL Northside School, Dongguan, China; 3 Wuhan No. 64 Middle School, Wuhan, China; 4 Wuhan Optics Valley No. 15 Primary School, Wuhan, China

**Keywords:** analytic hierarchy process, homeroom teacher, professional quality of life, qualitative research, gray statistics method

## Abstract

**Introduction:**

This study aimed to develop and validate three-level evaluation indicators for the quality of professional life of homeroom teachers.

**Methods:**

This study adopted an exploratory sequential mixed-methods design. First, we conducted semi-structured interviews with 143 homeroom teachers in China and analyzed 120 excerpts from published literature using NVivo 10. Next, 72 experts (elementary and secondary school principals) assessed and compared the evaluation indicators. We conducted statistical analysis using the Gray Statistical Method and AHP (Analytic Hierarchy Process) Calculation Method. Finally, we surveyed 661 teachers, 730 principals, and 792 teachers from Taiwan and verified the validity of the evaluation indicators using quantitative analysis methods.

**Results:**

In the AHP Calculation Method, when the order of the judgment matrix is greater than 2 and the CR (Consistnency Ratio) is far less than 0.1, the matrix is consistent, and weights can be further calculated. The survey results indicated that the overall occupational quality of life of head teachers was moderately low, with a score of 2.81(3 is the median for each question.), and the occupational quality of life of the sample was significantly lower than the median (3 is the median for each question.). The data obtained from the homogeneity reliability (Cronbach’s alpha) test of the questionnaire were 0.79, indicating good reliability. However, the homogeneity reliability of the Taiwan sample was unsatisfactory.

**Conclusion:**

By constructing and validating an evaluation indicator system for Chinese homeroom teachers’ professional life quality, this study provides standardized tools and empirical evidence to diagnose their professional predicaments, optimize school management, inform educational policy formulation, and advance relevant research—while introducing a scientifically weighted, employee-centered index with theoretical and practical policy significance for allowance systems and professional development programs.

## 1 Introduction

National statistical data and policy documents highlight critical challenges faced by Chinese homeroom teachers, with workload intensity, large class sizes, and inadequate remuneration creating interconnected pressures. According to the 2025 National Workload Quantification Standards, homeroom teachers working in large classes (≥46 students) work 8–10 weekly standard teaching hours, 14–25% more than regular subject teachers, yet non-teaching tasks (administrative reporting, parent communication, ideological education) consume over 70% of their working hours, leading to 1–2 h of daily overtime and leaving <30% of their time for core pedagogy (The Ministry of Education, 2025; Liu and Pan, 2025).

The 2025 National Education Statistics Report notes that 73% of elementary and 66% of junior high classes exceed 46 students, with national average student–teacher ideal ratios at 16.33:1 (elementary) and 12.64:1 (junior high); however, urban classes average 42–45 students. Meanwhile, 12% of rural teachers manage multi-grade classes, straining to provide individualized instruction (National Bureau of Statistics of China, 2025; China Institute of Education Sciences, 2025). Financially, despite the 2025 raise in monthly allowances for homeroom teachers to a national minimum of CNY 700 (≈USD 97, up from 500 to 600 CNY pre-2025), 68% of teachers still perceive that they are receiving “insufficient monetary recognition,” reflecting persistent remuneration disparities relative to workload (Ministry of Finance & Education, 2025; Chinese Society of Education, 2025). These data underscore a systemic imbalance: Homeroom teachers, burdened by dual roles as educators and student welfare administrators, face compounded pressures from large classes and inadequate compensation, threatening educational quality and retention.

Professional quality of life (PQoL) is one component of quality of life and serves as an indicator of employees’ occupational status. Carshaw, an American psychologist, defined PQoL as an employee’s sense of their physical and mental health at work (Ramlall and Sheppeck, 2007). Improved PQoL can lead to social and economic benefits (Todaro-Franceschi, 2024). Walton (1975) noted that the term PQoL is used in the context of the neglected humanist values of an industrial society and obsession with the pursuit of industrial productivity and economic growth, adding that focusing on employees’ PQoL can protect such values.

The concept of PQoL began gaining attention in the 1960s, when organizations, employees, and society realized that material returns alone could not satisfy employees’ needs or motivate them to work more efficiently (Galbraith, 2010). Consequently, scholars began to shift their focus toward research on PQoL. This line of research is based on the belief that employees are an organization’s most valuable resource and that treating them with dignity fosters sincerity, responsibility, and a strong sense of duty (Kagan and Melamed-Biran, 2024). Existing research on PQoL has mostly been carried out from the employee perspective. Studies conducted from the organizational perspective tend to incorporate PQoL (Luo et al., 2013) into broader investigations of work and life-related factors within the organization. Notably, most research on PQoL in China began to emerge after the 1980s (Zhao et al., 2010).

Most studies on PQoL have viewed it from organizational (Sirgy, 2022), employee (Sirgy, 2024), and societal (Kiernan and Knutson, 1990) perspectives. Although various definitions of “PQoL” exist, scholars generally regard it as a multi-dimensional concept encompassing factors such as job satisfaction, job pressure, work input, organizational support, career development, working environment (Agustina et al., 2024), work content (Baba and Jamal, 1991), and work–life balance (Farber et al., 2023). Other factors include salary and welfare (Swamy et al., 2015), career development opportunities (McDonald and Hite, 2023), and fair labor practices (Myhill et al., 2023). Collectively, these factors shape the general perceptions and evaluations of employees’ PQoL (Levine et al., 1984).

However, research on PQoL faces several challenges. Given the multidimensional nature of this concept, obtaining reliable data to support meaningful conclusions requires robust study designs, large-scale questionnaires or interviews, and effective data analysis procedures. In this context, ensuring sample representativeness and data quality is crucial. Additionally, evaluations of PQoL must effectively strike a balance between subjective experiences—such as job satisfaction (Salahat and Al-Hamdan, 2022) and happiness (Murgaš et al., 2022)—and objective indicators—such as work environment (Monroe et al., 2020) and salary level (Todaro-Franceschi, 2024; Stamm, 2010). Furthermore, research needs to capture and analyze how PQoL is affected by economic, social, technological, and other factors (Wu et al., 2010; Ramkissoon, 2023). Researchers need to account for differences in how PQoL is understood and valued across countries, regions, and industries, avoiding one-size-fits-all conclusions (Babapour et al., 2022). They must also ensure the security and privacy of collected data adhering to ethical review procedures and operational standards. Research should not only identify problems but also propose effective interventions and evaluate their practical outcomes. Additionally, given the complexity of professional-quality-of-life research and intersections with multiple disciplines, such as psychology, sociology, economics, and management, interdisciplinary cooperation is necessary. By addressing these challenges, researchers can more comprehensively and accurately evaluate PQoL, ultimately clarifying and contributing to its improvement (Todaro-Franceschi, 2024).

An insightful yet unexplored area of research in this field is the PQoL of homeroom teachers in China. In the Chinese education system, homeroom teachers play a unique role—they not only deliver subject knowledge but also guide students’ spiritual growth (Feng and Li, 2016). Along these lines, a homeroom teacher must be attentive to students’ overall development, including their outlook on life, values, physical and psychological health, and moral and esthetic sensibilities (Tahira and Haider, 2020). Homeroom teachers serve as educators, managers, and coordinators; they monitor students’ academic progress and address issues related to students’ mental health, moral development, and daily concerns (Maftuhah and Firdaus, 2024). Notably, homeroom teachers also provide psychological support, especially given the rise in student psychological problems due to modernization. In particular, they work to identify students’ psychological problems and help resolve them by providing emotional support and guiding students through the complexities of adolescence (Putri and Dasalinda, 2023). Moreover, homeroom teachers play the roles of fathers, mothers, brothers, and friends in the Chinese education system, which is rarely seen in other countries. In other words, they oversee all aspects of students’ school life, including their emotional well-being, and try to educate them in every possible way (Gu, 2015). Homeroom teachers in China also serve as a bridge between home and school. They regularly communicate with parents to provide feedback on students’ learning and behavior and gain insights into students’ home environments to better educate and guide them (Shi and Leuwerke, 2010). Accordingly, they may meet with parents, including participating in home visits, and engage parents in daily communication (Maftuhah and Firdaus, 2024).

To fulfill these roles, homeroom teachers organize various extracurricular activities, class meetings, and community services to help students develop their moral, intellectual, physical, esthetic, and labor capacities (Ye et al., 2023). Additionally, they develop and employ effective classroom management strategies (Shi, 2021); for example, they are responsible for formulating class rules and regulations, training class cadres, organizing rich and colorful class activities, and creating a good classroom atmosphere and study style (Sapir and Mizrahi-Shtelman, 2024). Through careful design and effective management, they foster a united, friendly, and positive classroom environment. In sum, Chinese homeroom teachers take on uniquely comprehensive and diverse roles—educating students, managing classrooms, facilitating communication between home and school, and providing psychological counseling (Treska and Treska, 2016). Given that they not only impart knowledge but also cultivate students’ moral character, ability, and physical wellness (Chen and Zhao, 2022), homeroom teachers in China are broadly responsible for promoting students’ all-around development.

The profession of homeroom teacher has historically been special in China. Since the introduction of the homeroom teacher system, homeroom teachers have assumed the responsibility of preserving cultural values and helping every child realize their social potential (Treska and Treska, 2024; Alia Putri et al., 2024). Notably, the homeroom teacher is the most central member of the classroom. As such, homeroom teachers must maintain both physical and mental health to effectively fulfill their duties in caring for their students. If the PQoL of homeroom teachers is poor, it will directly affect children’s academic performance and mental well-being. In some cases, homeroom teachers may even have hidden mental problems that could pose a direct threat to the physical safety of children in the classroom (Baeriswyl et al., 2021; Chen and Zhao, 2022). Homeroom teachers engage in intellectual and emotional labor and may care more about the returns on their efforts than other professionals (Feng and Li, 2016). As intellectuals, homeroom teachers are attuned to their spiritual needs and PQoL (Sun, 2012; 2016). Thus, assessing and intervening in the PQoL of homeroom teachers is necessary for their professional development, the healthy development of the next generation in China, and the progress toward realizing the Chinese dream.

Research on occupational quality of life still prioritizes performance over quality management. This tendency stems largely from the prevailing utilitarian perspective in management studies, which equates effective management with maximizing output and achieving better outcomes. However, this view is not entirely accurate. All labor results can be quantified and observed, especially in education, where teachers’ influence on students often unfolds gradually and in less tangible ways. While student academic performance is the most visible indicator, many essential aspects of a teacher’s contribution to student development cannot be immediately detected and are therefore only observable in the long term. Overemphasizing short-term performance, reducing educators to mere tools, will ultimately lead to their passivity and diminished creativity. Such a perspective would be deeply detrimental to the education sector.

Accordingly, evaluating homeroom teachers’ PQoL is necessary to improve schooling (Wang, 2021). Given homeroom teachers’ key roles in classroom management and student education, their PQoL directly affects the quality of education and student development (Engreini, 2019). For example, by studying their PQoL, we can identify the factors affecting their work-related pressures, needs, and outcomes. Based on this, measures to optimize the allocation of educational resources can be developed, improving the overall efficiency and effectiveness of the education system. Therefore, insights into homeroom teachers’ PQoL are useful for educational administrators; in particular, administrators may apply these insights to make reasonable decisions or formulate more effective policies and support systems that promote homeroom teachers’ professional development, mental health (Yang et al., 2009), and overall status (Siddikov and Mahkamova, 2022). Meanwhile, given that the PQoL of homeroom teachers is closely related to their students’ attention and patience, research in this area can also be applied to improve the relationship between teachers and students. Finally, investigating the PQoL of homeroom teachers can shed light on their psychological struggles. Because the mental health of homeroom teachers directly affects their students’ emotional and psychological well-being (Jimmieson et al., 2010), addressing these teachers’ PQoL is a necessary step toward creating a healthier learning environment (Damásio et al., 2013).

In recent years, with the deepening of education reform, homeroom teachers’ PQoL has become a focal point of attention across various sectors. However, research on the PQoL of homeroom teachers is scarce. Although it is well-known that homeroom teachers play the most significant role in teaching groups and have the strongest educational influence on students, existing research is largely focused on their work performance and work level, with limited attention to their PQoL. Prior research has predominantly prioritized performance or managerial perspectives while neglecting the living and working conditions of homeroom teachers—a critical oversight within the discourse on PQoL. The proposal of this study, constructing an employee-centered, analytic hierarchy process (AHP; Analytic Hierarchy Process)-weighted evaluation index, should be positioned as a direct response to this research gap.

This study holds dual significance: theoretically, it advances PQoL research by foregrounding the experiences of a understudied demographic, thereby expanding the theoretical scope beyond conventional performance metrics; practically, it offers empirically grounded insights to inform educational policies aimed at ameliorating homeroom teachers’ work environments, addressing systemic imbalances between workload, class management, and well-being. This study sought to construct a scientifically grounded and reasonable index system to comprehensively evaluate Chinese homeroom teachers’ PQoL. Additionally, the study sought to apply this index to assess the current PQoL of homeroom teachers in China, analyzing factors such as their working environment, work pressures, career development, salary, welfare, work autonomy, and social support. Based on the findings, targeted suggestions are proposed for improving the quality of life of Chinese homeroom teachers. This research not only deepens our understanding of the working conditions and needs of homeroom teachers but also provides valuable insights for the formulation of educational policies aimed at enhancing the overall PQoL of homeroom teachers.

## 2 Materials and methods

### 2.1 Establishment of the evaluation index

#### 2.1.1 Study design and implementation

This study adopted an exploratory sequential mixed-methods design to develop a new evaluation index for the PQoL of homeroom teachers in China. First, qualitative data were collected through semi-structured interviews and a literature review to construct the initial framework. Second, quantitative input from a panel of experts was used to refine the indicators and determine their weightings. Most tools currently available for measuring the PQoL of homeroom teachers in China have been developed from the manager’s perspective, focusing on methods and effectiveness in managing class teachers rather than addressing their experiences. Previous studies have emphasized the need to describe teachers’ experiences rather than scientifically evaluating their PQoL (Stamm, 2010). Some scholars have proposed a manual for the PQoL scale and a few indicators based on input from front-line homeroom teachers, which have been utilized to collect small amounts of data. However, scales used in studies on the PQoL of homeroom teachers remain insufficient (Li and Sun, 2017).

Conducting professional-quality-of-life research from an employee perspective has natural advantages. First, it allows individuals to evaluate their own PQoL. Second, PQoL includes subjective experiences and feelings that can be best captured through self-evaluation. Such information can only be known to the homeroom teachers themselves. An evaluation scale that accounts for this information could provide for a more comprehensive evaluation. Third, in China, most homeroom teachers do not communicate directly with the school principal and instead report to intermediate managers, which makes it difficult to objectively evaluate their PQoL from the principal’s perspective. Therefore, in China, evaluating professional life quality from the perspective of employees using evaluation indicators based on data collected from front-line employees is more appropriate. Accordingly, this study developed a set of indicators to scientifically evaluate the PQoL of front-line homeroom teachers. Compared with existing tools, the evaluation tool developed in this study has the following advantages: (1) it was developed through scientific research procedures, making it scientifically robust; (2) it is based on the employee perspective, capturing the actual situation of homeroom teachers; and (3) it aligns more closely with the developmental context of Chinese homeroom teachers.

#### 2.1.2 Study phases

This study was conducted in three phases. The first phase involved the development of the index framework through qualitative research. Specifically, we used NVivo 10 to analyze recordings of semi-structured interviews with 143 homeroom teachers (aged 23 to 60 with teaching experience ranging 0–40 years) as well as relevant excerpts from the “Education Life” column in the last 2 years of the *Friend of the Homeroom Teacher* magazine. Founded 40 years ago, the magazine features articles written by elementary and secondary school homeroom teachers. Over 100,000 copies of the magazine are sold annually. The content of this magazine primarily focuses on teaching pedagogy and classroom management practices among Chinese homeroom teachers; the author of the column is a classroom teacher in China.

The specific analysis process was as follows. First, we (the authors of the current research) listened to the recordings from the interviews and transcribed them based on open coding principles, followed by automatic coding. Subsequently, we held repeated discussions on the preliminary coding results until a clear outline emerged, from which we developed a preliminary diagram. In the second phase, we selected the indicators for the index using the whitening function of gray statistics to determine the specific measurement indicators. In the third phase, 72 experts (renowned homeroom teachers who were selected from across China; we call them outstanding homeroom teachers) were invited to compare and evaluate the indicators. Using the AHP, we analyzed the consistency of the data, determined the weightings of each dimension of PQoL, and optimized the tool’s effectiveness. The three phases of the research were progressive and coherent and, thus, scientifically robust. The AHP is a fuzzy mathematical evaluation method widely used in decision-making optimization and inference evaluation. Notably, the evaluation method derived through the AHP is more scientifically robust and effective than the evaluation methods obtained through factor analysis (Yang, 2015). Unlike traditional factor analysis, the AHP can incorporate expert evaluation to determine the weight coefficients of each index, making the final indicators of the tool for measuring PQoL more scientifically grounded. Furthermore, evaluations of PQoL may vary by teacher characteristics, such as age, years of teaching experience, region, and school year, and the AHP can be applied to tailor evaluation methods for PQoL across these diverse groups.

During the data collection phase, the main interview team comprised five members, each holding either a master’s or doctoral degree. Before the interviews, the team leader conducted multiple pre-interview sessions to ensure that all members were thoroughly familiar with the interview protocol before formally initiating data collection. In the coding and initial theory formation stage, the research group primarily consisted of four members: two doctoral psychologists, one researcher with a master’s degree, and one researcher with a doctoral degree in education. The entire process spanned approximately 2 months. Based on the literature review, the team designed a semi-structured interview guide that included explicit self-report and projective sections. The interview guide was divided into three parts: The first part covered basic demographic and professional information about the homeroom teachers; the second explored their understanding of the PQoL, perceived influencing factors, and evaluation criteria; the third introduced a projective test in which participants read a story and evaluated the PQoL of the outstanding homeroom teachers, Mr. Liu, attempting to explain the evaluation criteria. During the coding phase, team members first independently coded approximately 30 transcripts using NVivo 10. After reaching a consensus on the coding method, the remaining data were automatically coded to obtain information on nodes, reference points, and sources of materials.

#### 2.1.3 Establishing a framework for qualitative research

Based on a literature review, the research team designed explicit self-report instruments and prepared semi-structured interviews. The interviews were conducted with 143 randomly selected homeroom teachers from 14 elementary and secondary schools across Hubei province. Each interview was recorded and lasted approximately 20 min on average. One researcher facilitated the interviews, while the other assisted by managing the recording process. The interviews were transcribed into 143 interview texts, which served as the primary source of data for qualitative analysis.

**Figure 1:**
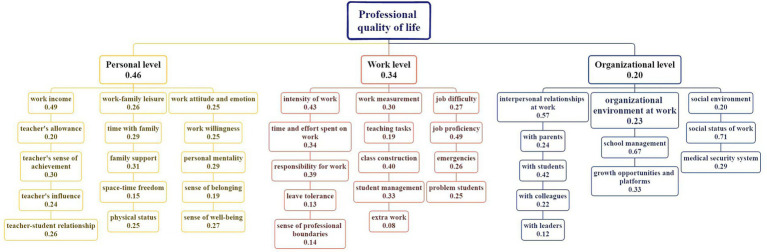
Three-level index system for the evaluation of the professional life quality of homeroom teachers.

The second source of data was the “Education Life” column from the *Friend of the Homeroom Teacher* magazine, which mainly focuses on the work life of elementary and secondary school homeroom teachers. We analyzed 120 excerpts from this column published within the past 2 years. Combined with the 143 interview transcripts, this yielded a total of 263 data sources for qualitative analysis. The qualitative analysis (conducted between 2022 and 2024) was guided by grounded theory (Chen, 2015), which is a widely used method in the Chinese social sciences and is considered one of the most powerful and influential approaches in qualitative research (Chen, 1999). In its early development, grounded theory was based on the assumption that there is an objective external law and theoretical framework that scientists must identify through objective and neutral observation and data analysis. Therefore, in research, social scientists must attempt to maintain a neutral position, objectively analyze data, and apply theory (Sun et al., 2018). To conduct the analysis, NVivo 10, an auxiliary tool for scientific research, was used. Specifically, it was used to analyze the frequency of words and automatically sort them. After coding and comparative classification, key concepts were extracted to develop a preliminary theoretical framework for the index, as shown in Figure 1.

#### 2.1.4 Development of the index

Based on the three-level indicators comprising the preliminary framework, an expert indicator evaluation questionnaire was designed to screen the index. A total of 109 homeroom teachers (aged 36–45 with an average teaching experience of 17.6 years) participated in the screening process. This group included nationally recognized homeroom teachers and outstanding teachers from the Hubei Province, including outstanding homeroom teachers. Their primary objective was to determine the importance of the proposed indicators for evaluating the PQoL of homeroom teachers and to design open-ended questions to allow participants to put forward their ideas. Of the 109 homeroom teachers, 55 were from urban schools, 42 from township and rural schools, and 12 from suburban areas. The teaching age (referring to their years of experience in teaching) of the homeroom teachers was relatively average. Among the participants, 49 were men and 60 were women. The survey did not include questions related to personal privacy, and all responses were submitted anonymously.

**Figure 2:**
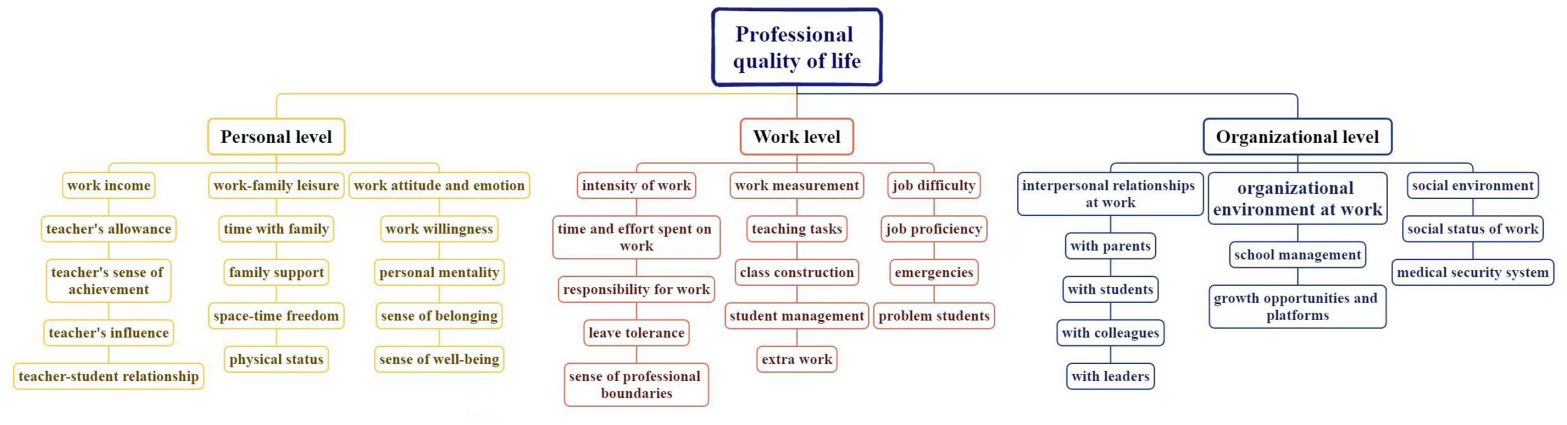
Overall sample analysis plot of the professional life quality of homeroom teachers.

Data recovery was conducted by performing a computer-based importance analysis. For this analysis, we invited a professional homeroom teacher with expertise in computing, based on the gray statistics method. To develop the homeroom teacher PQoL evaluation index system, we divided PQoL into three categories based on the structure of the gray function for various grades (Tian, 2012). We calculated the importance of each evaluation index by determining whether it had a “high” or “low” decision vector value, attributed importance to the highest of the three vector values, and eliminating the “low” values as unimportant to the index. The overall sample analysis plot is demonstrated in Figure 2. After further analysis of different sample groups, including variables such as sex, region, age, and years of teaching experience, we found only minor differences across a few indicators. Consequently, the practice of subdividing by demographic variables was discontinued, and the overall indicators were used directly.

#### 2.1.5 Using the AHP to determine the weight of the indicators

The AHP is a fuzzy mathematical evaluation method widely used to optimize decision-making and inference evaluations (Yang, 2015). Unlike traditional factor analysis, the AHP relies on expert evaluations to determine the weight of each indicator within the index, considering factors such as age, years of experience, region, and grade level. Therefore, it can be applied to develop different evaluation methods for assessing the PQoL indicators of homeroom teachers across all levels of education.

After obtaining the evaluation index system presented in Figure 1, further determining the weight coefficient of each index was necessary. According to fuzzy mathematical theory, the weight coefficient can be determined based on the evaluation of a limited number of experienced experts, with each expert’s evaluation contributing to the coefficient’s formation. During this expert evaluation stage, we employed a one-to-one on-site interpretation and recovery method. This phase was completed between September and November 2023. The experts were homeroom teachers participating in a forum held in Chengdu, Sichuan Province, and homeroom teachers from third- and fourth-year classes in Hubei Province (These participating teachers were selected from outstanding homeroom teachers nationwide). The training sessions were held in Chengdu (Sichuan province) and Wuhan (Hubei Province). According to fuzzy mathematical theory, the consistency of the matrix should be tested before calculating weight coefficients. In the AHP, a matrix is considered consistent—and thus, the weight can be calculated (Yang, 2015)—when the order is greater than 2 and the CR (Consistnency Ratio) is less than 0.1. If these conditions are not met, the values should be adjusted according to the exponential prompts.

This study adhered to the minimal adjustment principle: Only one adjustment is allowed to meet the consistency requirements, with a maximum of two values being adjusted. If the requirement cannot be satisfied within these limits, the matrix is discarded and not included in the calculation. The research team spent approximately a week testing each matrix. The eligible data were then entered into SPSS 11.5 to calculate their average. Next, the average value of the total sample was transformed into a new matrix, which was inserted into the formula for calculating the weight coefficient to obtain the weight index for the overall sample. No significant differences based on sex, region, grade level, or years of experience were found in the mean values of the homeroom teachers. Thus, the matrix of the overall sample was deemed valid, and the obtained weight indices were applicable to all homeroom teachers. Tables 1–4 list the weight coefficients for each indicator across the three-level index (In Table 1, A represents the primary indicator at the individual level, B represents the primary indicator at the work level, and C represents the primary indicator at the organizational level. The sub-tables a1, a2, and a3 represent three secondary indicators at the individual level; b1, b2, and b3 represent three secondary indicators at the work level; and c1, c2, and c3 represent three secondary indicators at the organizational level).

**Table 1:** AHP analysis: individual-level consistency ratio and indicator weights.

A	a1	a2	a3	Weight coefficient
a1	1	2.09	1.75	0.49
a2	0.48	1	1.14	0.26
a3	0.57	0.88	1	0.25

CI = 0.0053 λ = 3.011 CR = 0.009.

**Table 2:** AHP analysis: work-level consistency ratio and indicator weights.

B	b1	b2	b3	Weight coefficient
b1	1	1.63	1.35	0.43
b2	0.61	1	1.31	0.30
b3	0.74	0.76	1	0.27

CI = 0.0116, λ = 3.023, CR = 0.02.

**Table 3:** AHP analysis: organization-level consistency ratio and indicator weights.

C	c1	c2	c3	Weight coefficient
c1	1	2.76	2.66	0.57
c2	0.36	1	1.26	0.23
c3	0.38	0.79	1	0.20

CI = 0.0012, λ = 3.0084, CR = 0.0072.

**Table 4:** AHP analysis: index consistency ratio and indicator weights.

Category	A	B	C	Weight coefficient
A	1	1.48	2.15	0.49
B	0.68	1	1.94	0.34
C	0.47	0.52	1	0.20

CI = 0.0047, λ = 3.0095, CR = 0.00815.

#### 2.1.6 Validation of evaluation indicators

In September 2024, 730 elementary and secondary school outstanding homeroom teachers from various regions of Hubei Province were surveyed using a random cluster sampling method. In this sample, the average class size in mainland Chinese schools was approximately 53 students. The following results are based on the weighted dimensions of three-level indicators. For clarity, data that require reverse scoring have been processed in the charts. Higher values indicate better occupational quality of life. The first four data points in Table 5 represent the validity of the indicators, while the subsequent data points represent reliability. Table 5 shows that for mainland outstanding homeroom teachers, using secondary indicators as tertiary indicators, the calculated reliability and validity indicators all meet the requirements.

**Table 5:** Reliability and validity indicators of quality of life and its dimensions.

Region	R_t2*3_	R_a2*3_	R_b2*3_	R_c2*3_	R’_t2*3_ (Homogeneity reliability)
Mainland China	0.62***	0.63***	0.55***	0.57***	0.79***
Taiwan	0.04	0.08^*^	0.10^***^	−0.03	0.06

*** indicates *p* < 0.001, highly significant. Rt2*3 represents the correlation coefficient between secondary and tertiary indicators at the overall level; Ra2*3 represents the correlation coefficient between secondary and tertiary indicators at the individual level; Rb2*3, at the work level; Rc2*3, at the organizational level. The data in the first row come from Mainland China sample, and the data in the second row come from the Taiwan, China sample.

Further analysis was conducted on issues related to the PQoL for outstanding homeroom teachers in Mainland China. Table 6 presents the correlation coefficients and significance levels among PQoL, work efficiency, and job responsibility. The results indicate that PQoL is significantly positively correlated with quality of life, work efficiency, and job responsibility. The data indicate that the evaluation indicators developed in this study yielded strong reliability and validity, with good discriminatory power.

**Table 6:** Correlation between quality of life and other variables.

Category	Quality of life	Work efficiency	Dedication to work
Quality of life at work	0.44***	0.31***	0.14***

*** indicates *p* < 0.001, highly significant.

#### 2.1.7 Cross-cultural adaptability of the instrument

To verify the cultural appropriateness, the research instrument was applied to survey class advisors in Mainland China and Taiwan. Although Taiwan and Mainland China share common cultural roots, there are many differences in the work content and methods of class advisors. For instance, elementary schools in Taiwan implement a system where the class advisor is responsible for the entire class. In September 2024, a stratified random sampling (by educational level) was conducted among 792 class advisors across the northern, southern, eastern, central, and offshore islands of Taiwan. The average class size in Taiwan is approximately 21 students per class. As shown in Table 5, for mainland Chinese class advisors, the reliability and validity indices calculated using secondary indicators as tertiary indicators met the required standards. However, for Taiwanese class advisors, the reliability and validity indices did not meet these standards. Therefore, this indicator system needs to be revised based on the actual cultural context to more accurately assess the PQoL for teachers in that culture.

## 3 Results

### 3.1 Professional quality of life of homeroom teachers in China

#### 3.1.1 Overall professional quality of life

We conducted a study with 661elementary and secondary school homeroom teachers selected through random cluster sampling. Within the sample, sex and grade level were roughly balanced. Most teachers worked in various regions of Hubei Province, including Ezhou, Huanggang, Huangshi, Jingzhou, Xiaogan, Suizhou, Xiangyang, and Wuhan. The results of the questionnaire (which included the concepts demonstrated in Figure 1 and was designed based on the indicators established above at the individual, work, and organizational levels) were analyzed using SPSS 19.0. The overall PQoL among the surveyed homeroom teachers was below moderate, with an average score of 2.81. The median value for each topic was 3. A one-sample t-test comparing the sample’s score to the median showed significant differences, indicating that the PQoL for the sample was significantly below the median. We also conducted an adaptability test, finding that the correlation between the secondary and tertiary indicators was 0.62 (significance level: 0.001). In terms of reliability, the homogeneity reliability test in the questionnaire yielded a value of 0.79. According to Dai et al. (2005), a correlation above 0.60 between two standardized tests indicates good reliability; thus, the index in this study is reliable and can be used as an effective evaluation tool.

### 3.2 Differences in professional quality of life

#### 3.2.1 Sex

Male homeroom teachers had significantly lower scores than female homeroom teachers in the dimensions related to work, family, and leisure. In the work attitude dimension, male homeroom teachers scored 2.61, while female homeroom teachers scored 2.80 (*p* = 0.003). In the dimension of emotions, male homeroom teachers scored 3.05, while female homeroom teachers scored 3.22 (*p* = 0.003). In the interpersonal dimension, male homeroom teachers scored 3.85, while female homeroom teachers scored 24.01 (*p* = 0.002). In the organizational environment dimension, male homeroom teachers scored 3.24, while female homeroom teachers scored 3.44 (*p* = 0.003). At the organizational level, male homeroom teachers scored 3.46, while female homeroom teachers scored 3.63 (*p* = 0.000). Female homeroom teachers tend to have better professional experiences than male homeroom teachers in education, which is stereotypically regarded as “women’s work” (Shen and Xiong, 2014).

#### 3.2.2 Marital status

Differences in marital status were significant in terms of the difficulties faced at work: Being married correlated with facing fewer difficulties at work, with a significance level of 0.002. As teachers age, they become more adept at negotiating conflicts between work and personal life and may even integrate the two with ease. Notably, marriage and family are regarded as the two most important factors affecting overall happiness. PQoL was highest for elementary school homeroom teachers, followed by middle school homeroom teachers and high school homeroom teachers.

#### 3.2.3 Grade level

In terms of grade level, significant differences were observed across the individual and organizational dimensions, but no differences were observed for the work dimension. The results for elementary school teachers were superior to those for middle school teachers, while those for middle school teachers were superior to those for high school teachers. This may be due to the lower pressure on elementary school teachers, with the highest PQoL observed among them, consistent with previous research (Hu et al., 2015).

#### 3.2.4 School location

In the organizational level dimensions, the PQoL was higher for rural homeroom teachers than for urban homeroom teachers. In the dimension of work difficulty, urban homeroom teachers faced more issues than rural homeroom teachers.

### 3.3 Factors affecting professional quality of life

#### 3.3.1 Years of work experience

Age, grade level, and years of experience impacted all dimensions of the work level. As homeroom teachers age, they may have less physical strength and energy to apply at work and may, therefore, evaluate their work intensity and workload more negatively. However, with age, they also gain more experience, which may make them feel like their work is becoming easier. The younger the homeroom teacher, the more inclined they are to think that their work is difficult. Age, years of experience, and organizational superiority were significantly negatively correlated (*r* = −0.093, *r* = −0.104, *p* < 0.05). Therefore, as homeroom teachers age, their management abilities improve, and they may become more comfortable with their work. This finding is consistent with existing studies (Luo, 2014).

#### 3.3.2 Number of students

The number of students was related to the intensity of the teacher’s work (*r* = −0.095, *p* < 0.05). The higher the number of students in the class, the higher the teacher’s work intensity; however, this impact was relatively small. Along these lines, previous research has shown that homeroom teachers with more than 50 students have higher rates of burnout (Yao and Zheng, 2016).

#### 3.3.3 General allowance

A significant positive correlation was observed between the teachers’ organizational superiority and their allowance (*r* = −0.09, *p* < 0.05), as well as work gain and return (*r* = −0.16, *p* < 0.001; see Figure 1 for the specific variables). As the closeness of homeroom teachers is a fixed value, the more they invest, the more they expect to gain. However, as homeroom teachers’ salaries are currently unsatisfactory, the correlation is negative. Since allowance is earned through work, these relationships are easy to understand. Notably, allowances were significantly positively correlated with organizational superiority but not with other dimensions, such as work attitude and emotions. One indicator of organizational superiority is the social status of the work, reflecting that organizational superiority is based on comparison. At present, the allowance system for homeroom teachers in China is outdated—the standard is too low and does not match the actual workload, creating a gap that needs to be addressed (Xue and Li, 2017).

#### 3.3.4 Work performance

As quality of life is a superior concept encompassing PQoL, it directly influences it. Our findings indicated that the higher the PQoL, the greater the work efficiency and the stronger the sense of work responsibility.

## 4 Discussion

### 4.1 Evaluation index

#### 4.1.1 Salary-related influencing factors: the dual role of material and spiritual indicators and their theoretical association

Homeroom teachers’ salary can be attributed to four factors: their allowance (Sossy, 2021), their sense of achievement (Bhai and Horoi, 2019), their influence, and the quality of the teacher–student relationship (Wanders et al., 2020). Of these four indicators, only the first is a material indicator; the other three are spiritual indicators. Notably, the weight coefficient revealed that the spiritual indicators have a significant impact on the PQoL of homeroom teachers (Rivkin et al., 2005), while the satisfaction of their material needs plays a greater role in their overall satisfaction (Makungu, 2021). This differential impact pattern of material and spiritual indicators both inherits and expands upon Lee (2018) research on teachers’ professional satisfaction. Lee also identified the dual dimensions of material and spiritual needs, but the present study further reveals the subdivided pattern that “spiritual indicators dominate PQoL” (Eirín- Nemiña et al., 2022), whereas “material needs dominate overall satisfaction” (Rudaleva et al., 2014). This may stem from the fact that PQoL focuses more on psychological experiences in specific professional domains(Admiraal and Røberg, 2023), while overall satisfaction covers all aspects of life, thus giving material needs higher weight (Rudaleva et al., 2014).

According to the two-factor theory, if an employee’s material needs are not met, they will not be satisfied with their PQoL; however, even if these needs are met, employees may not necessarily be satisfied with their PQoL. Meeting the needs captured by the spiritual indicators is necessary for ensuring professional satisfaction among employees (Miao and Xu, 2012). This is highly consistent with the core view of Herzberg et al.’s (1959) two-factor theory—that hygiene factors (e.g., material needs) can only eliminate dissatisfaction, while motivators (e.g., spiritual needs) are the key to generating satisfaction (Oyekunle, 2024)—confirming the applicability of this theory to the teacher group in educational contexts, especially highlighting the irreplaceability of spiritual motivation in homeroom teachers’ PQoL.

#### 4.1.2 Work evaluation indicators: work characteristics centered on high intensity and their group differences

The evaluation indicators of work are the characteristics of the work itself, including the intensity, measurement, and difficulty of the work (Saimi et al., 2024). Among them, work intensity was found to carry the largest weight. The work of the homeroom teacher is primarily reflected in the high intensity, which is reflected in the high responsibility of the role and the time and energy spent on the work (i.e., teacher job stress). Most of their workload is centered on class construction and student management; they have relatively few teaching tasks and administrative chores, but this relatively small workload still directly affects the evaluation of their PQoL (Zhao et al., 2021). This stands in sharp contrast to Liu et al.’s (2020) research on the work intensity of ordinary subject teachers: Ordinary teachers’ work intensity mainly stems from teaching tasks (e.g., lesson preparation, teaching, homework correction; Wilson, 1962; Wohlfart and Wagner, 2023; Te Braak et al., 2024), while homeroom teachers’ intensity is concentrated on class construction and student management (Putri and Dasalinda, 2023).

The root cause of this difference lies in the homeroom teacher’s role as the “primary person responsible for the class”—their core responsibility is students’ all-around development and class order maintenance rather than single-subject teaching (Fauziah et al., 2025; Li et al., 2023). Generally, then, homeroom teachers do not face significant work difficulties; with experience and skill, they can manage their everyday tasks with ease. When they do face difficulties, they are mainly related to student emergencies and other such problems. This aligns with Zhang (2014) conclusion that “teacher experience is negatively correlated with work difficulty,” but these results further emphasize that “student emergencies” are an exception (Papaya-Credo, 2025; Oved and Raichel, 2025). This is closely related to the homeroom teacher’s role as the “first responder to daily student management” (Raphaelli-Hirsch et al., 2025)—ordinary teachers rarely directly handle sudden issues such as student psychological crises or conflict mediation, so such difficulties are more unique to homeroom teachers (Liu and Barnhart, 1999).

#### 4.1.3 Organizational-level influencing factors: multidimensional environment centered on interpersonal relationships and their priority differences

At the organizational level, interpersonal relationships, organizational environment (Mulyana et al., 2021), and social environment (Dewi et al., 2021) were found to be key dimensions of PQoL. Regarding their interpersonal relationships at work, homeroom teachers may be affected by their relationships with students, parents, colleagues, and leaders (Bechter et al., 2023). In order of impact, the most important relationships are those with students (Zheng, 2021), followed by those with parents and colleagues. This priority ranking partially aligns with Pinkerton et al.’s (2023) research on elementary school teachers (both rank student relationships first), but there is a difference in the weight of colleague relationships: In Kim’s study, colleague relationships were equally as important as student relationships, while in this study, colleague relationships rank lower (Yablon and Itzhaky, 2013; Parks and McKay, 2023). This may be because homeroom teachers’ work relies more on direct interactions with students (Vlah et al., 2021), whereas ordinary teachers need to collaborate with colleagues to complete curriculum design, teaching discussions, and so on, thus having a higher dependence on colleague relationships (Chen et al., 2025).

In general, education leaders provide support for the work of homeroom teachers, but homeroom teachers usually do not view such support as an important factor influencing their PQoL. Therefore, the weight assigned to the relationship with leadership in the PQoL of homeroom teachers is relatively small (Jardí et al., 2022). This significantly differs from Parker et al.’s (2020) conclusion in corporate employee research that “leadership support is the primary predictor of professional satisfaction” (Guntuku et al., 2022). The underlying reason for this difference lies in the “flat interaction” characteristic of educational organizations: Homeroom teachers’ daily work directly faces students and parents, with a low frequency of indirect communication with leaders (Asis et al., 2025), whereas corporate employees’ performance goals and resource acquisition highly depend on leadership decisions, thus making leadership relationships more influential(Ansah et al., 2018).

#### 4.1.4 Key three-level indicators: dominant factors centered on teacher–student relationship and sense of achievement

Among the 31 three-level indicators, the top six were the homeroom teacher’s sense of achievement (Butler, 2007), the quality of interpersonal relationships with students (an indicator we constructed above; see Figure 1; Li et al., 2022), the great responsibility of work (Kostogriz, 2019), the influence of the homeroom teacher (McCroskey and Richmond, 2012), the quality of the teacher–student relationship (an indicator we constructed above; see Figure 1), and the time and energy spent on work (Jones et al., 2022). Among the six indicators, three belonged to the dimension of work gain and return at the individual level. The trade-off between pay and return remains the most fundamental dimension of PQoL. Notably, the teacher–student relationship appeared twice, as it represents both the objective and the result of the work. This dual attribute contrasts with Taylor and Kliestikova’s (2018) research, which treats the “teacher–student relationship” as a single “outcome variable.” The finding in this study may stem from the longitudinal tracking design: Homeroom teachers actively take “building good teacher–student relationships” as a goal in their daily work (objective; Heinla and Kuurme, 2024), and improved relationship quality in turn enhances their professional satisfaction (result; Veldman et al., 2013), thus presenting a dual role.

The homeroom teachers’ relationships with their students represent a key factor in shaping their PQoL. Other indicators are demonstrated in Figure 1; although they may not have a particularly large impact, they still influence the PQoL of homeroom teachers. Indeed, the absence of these indicators could have a particularly significant impact. This “significant impact of the absence of peripheral indicators” aligns with Hobfoll’s (2001) conservation of resources theory, which posits that “resource loss has a stronger impact than resource gain.” However, unlike Wang (2021) research, which focuses only on high-weight indicators, this study emphasizes the need to consider both primary and secondary indicators—homeroom teachers’ PQoL is a multidimensional system (Williams, 2012), and the absence of low-weight indicators (e.g., details of the organizational environment) may disrupt the system balance (Monroe et al., 2020; Ni et al., 2023; Toropova et al., 2020), which has important implications for comprehensive intervention.

### 4.2 Method characteristics

Our evaluation index for homeroom teachers’ PQoL, obtained through the AHP, differs from previous evaluation methods (Gawlik, 2019). Earlier methods primarily employed factor analysis to obtain evaluation indices, but these methods did not include specific weight allocations for the indicators. Contrarily, the AHP allocates weights across indices, with each weight being calculated only after a consistency test. The indices can be adapted to each situation and continuously adjusted, as needed, which is a notable advantage of using the AHP over factor analysis.

While the AHP-based evaluation index demonstrates methodological strengths, this study acknowledges potential limitations that warrant consideration. A key constraint is the regional concentration of the sample, which was primarily drawn from Hubei Province. Educational systems, cultural contexts, and administrative practices vary significantly across different regions, and thus the weight allocations and index priorities derived from Hubei samples may not fully capture the nuances of homeroom teachers’ PQoL in other provinces or municipalities. This regional focus risks overgeneralization of the results, as factors such as local policies on teacher welfare, parental involvement patterns, and student demographic characteristics could influence the relative importance of evaluation indicators. Future research should aim to replicate this analysis with geographically diverse samples to enhance the external validity of the findings.

### 4.3 Policy directions

The above findings offer valuable insights into best practices for improving the PQoL of Chinese homeroom teachers. First, updating the homeroom teacher system should be prioritized; in particular, the homeroom teacher allowance system, which has not been updated for 29 years, should be reviewed. Teachers’ compensation must be aligned with their performance evaluations to better satisfy their material needs (Allegretto, 2022; Biasi and Sarsons, 2021). Second, homeroom teachers should be given more opportunities for promotion (Berdaliyeva, 2023) and professional development, enabling them to reach their potential (Fischer et al., 2022); these opportunities should be especially extended to male teachers. Finally, support systems should be established according to the local conditions of different regions and student needs to reduce the professional uncertainty and challenges homeroom teachers currently face.

## 5 Conclusion

This study aimed to construct a comprehensive evaluation index system for the PQoL of homeroom teachers in China using the AHP with three sequential stages: qualitative framework development, indicator screening via gray statistics, and weight determination through expert consensus. By integrating qualitative research, gray statistical analysis, and AHP, this research sought to address the limitations of existing evaluation tools, which often lack systematic weighting and regional specificity.

The study yielded several notable results. First, the overall PQoL score of the sampled homeroom teachers (*n* = 661) was 2.81 (on a 5-point scale), significantly below the midpoint of 3 (*p* < 0.05), indicating moderate-to-low professional life quality. The evaluation tool demonstrated good reliability, with a Cronbach’s *α* of 0.79, exceeding the threshold of 0.60 for acceptable internal consistency. Second, the top six weighted indicators (out of 31 tertiary indicators) were: (1) sense of achievement, (2) quality of teacher–student relationships, (3) work responsibility, (4) teacher influence, (5) teacher–student relationship dynamics, and (6) time and energy investment in work. These indicators highlighted the primacy of psychological fulfillment and student interactions in shaping homeroom teachers’ PQoL. Third, AHP analysis confirmed that spiritual indicators (e.g., sense of achievement) carried greater weight in PQoL evaluation than material indicators (e.g., allowance), aligning with Herzberg’s two-factor theory, which emphasizes motivators over hygiene factors for satisfaction.

Theoretically, this research advances PQoL assessment by integrating grounded theory (for framework development), gray statistics (for indicator screening), and AHP (for weight calculation). This tripartite methodology addresses limitations of traditional factor analysis by providing objective weighting coefficients and ensuring indicator relevance through systematic qualitative and quantitative validation. Practically, the validated evaluation tool offers actionable insights for educational administrators: It can guide targeted interventions such as optimizing allowance policies (to address material needs as a foundational hygiene factor) and designing professional development programs focused on enhancing teacher-student relationships and sense of achievement—key spiritual motivators identified in the study.

Despite its contributions, this study has limitations. First, the sample was geographically concentrated in Hubei Province, limiting generalizability to other regions with distinct educational contexts. Second, cross-cultural validation efforts in Taiwan were unsuccessful, likely due to differences in educational systems and teacher roles, underscoring the need for context-specific adaptation of the evaluation framework.

To address these limitations, future studies should: (1) Conduct nationwide validation with samples from diverse provinces to enhance external validity. (2) Implement longitudinal designs to track changes in PQoL over time, particularly in response to policy interventions (e.g., allowance reforms). (3) Explore cross-cultural adaptation in other Chinese regions or international contexts (e.g., comparative studies with homeroom teachers roles in East Asian education systems) to refine the framework’s cultural sensitivity.

In conclusion, this research provides a robust methodological and empirical foundation for understanding and improving homeroom teachers’ PQoL. By bridging theoretical innovation with practical application, it paves the way for evidence-based policies to support this critical educational workforce.

## Data Availability

The datasets presented in this study can be found in online repositories. The names of the repository/repositories and accession number(s) can be found in the article/supplementary material.
